# Integrative Analysis of Cancer-Related Signaling Pathways

**DOI:** 10.3389/fphys.2013.00124

**Published:** 2013-06-04

**Authors:** Thomas Kessler, Hendrik Hache, Christoph Wierling

**Affiliations:** Systems Biology Group, Department Vertebrate Genomics, Max Planck Institute for Molecular Genetics, Berlin, Germany

**Keywords:** modeling of signaling pathway, cancer gene expression, expression signature, sample stratification, microarray analysis

## Abstract

Identification and classification of cancer types and subtypes is a major issue in current cancer research. Whole genome expression profiling of cancer tissues is often the basis for such subtype classifications of tumors and different signatures for individual cancer types have been described. However, the search for best performing discriminatory gene-expression signatures covering more than one cancer type remains a relevant topic in cancer research as such a signature would help understanding the common changes in signaling networks in these disease types. In this work, we explore the idea of a top down approach for sample stratification based on a module-based network of cancer relevant signaling pathways. For assembly of this network, we consider several of the most established cancer pathways. We evaluate our sample stratification approach using expression data of human breast and ovarian cancer signatures. We show that our approach performs equally well to previously reported methods besides providing the advantage to classify different cancer types. Furthermore, it allows to identify common changes in network module activity of those cancer samples.

## Introduction

Deregulation of growth regulating signaling pathways contributes to an extensive alteration of cellular physiology and confers excessive proliferation properties to cancer cells (Hanahan and Weinberg, 2000, 2011). Such perturbation of a cell’s signaling system can for example be caused by mutations in upstream signaling proteins (Gu et al., 2007), within coactivators of a signaling cascade (Björklund et al., 2007), components of the downstream signal transduction cascade (Irby et al., 1999; Bachman et al., 2004; Garnett and Marais, 2004; Carpten et al., 2007), or loss of function mutations of negative pathway regulators (Bonneau and Longy, 2000). Frequently, upregulated gene expression of growth factor ligands and/or their receptors such as amplification and subsequent overexpression of ERBB2 (Yarden and Sliwkowski, 2001) or Hedgehog (Ehtesham et al., 2007) can contribute to tumor growth. Oncogenic pathway activation can then lead to secondary transcriptional deregulation of factors that confer feedback within the same pathway, such as the dual specific phosphatase (DUSP) feedback inhibitors (Owens and Keyse, 2007). Furthermore, recent work suggests that the mRNA expression pattern of signaling components in cancer cells shows a certain plasticity in response to drug treatment, a mechanism that might help cancer cells to evolve into drug resistant cancer cell clones (Johannessen et al., 2010; Nazarian et al., 2010). These and other studies are consistent with the idea that mRNA expression levels of receptor coupled signaling pathway components might directly reflect the activity status of the respective pathway in a given tumor sample.

Tracking deregulation of signaling events in a given cancer sample is of great clinical interest as certain cancer types might be treatable with drugs developed to specifically inhibit receptor coupled upstream signaling events thereby allowing personalized intervention schemes. Thus, methods of cancer sample classification have been developed that in principal are based on the mRNA expression status of a set of discriminatory signature genes. For example (Bild et al., 2006) used the expression pattern of experimentally derived pathway signature gene sets for successful stratification of breast cancer patient samples. Other groups defined discriminatory gene signatures for breast or ovarian cancer based on statistical analysis of the expression profiles of a given set of cancer subtypes (van’t Veer et al., 2002; Sorlie et al., 2003; Wang et al., 2005; Denkert et al., 2009; Mok et al., 2009) or by defining a gene signature common to metastatic cancer samples (Rhodes et al., 2004). In two exemplary studies, the mRNA expression data of multiple patients was used as training sets to derive patient group intrinsic differences of mRNA expression. These defined gene signatures could then be used for successful stratification of independent test groups of patients and may be used as clinically relevant indicators of cancer status (van’t Veer et al., 2002; Sotiriou et al., 2006). While those gene signatures provide indirect measures for pathway activity in a sample set, more recent work has integrated gene-expression profiles with molecular-pathway or protein–protein interaction information (Peri et al., 2003; Chuang et al., 2007; Chowdhury and Koyutürk, 2010; Dao et al., 2010; Eddy et al., 2010). For example, Eddy and coworkers used 250 BioCarta pathways from MSigDB for improving their classification of cancer samples. The method of Chowdhury and Koyutürk that relies on matched tumor and control samples has been improved to successfully predict the p53 status in breast cancer samples and liver metastasis in colon cancer using networks of STRING derived protein–protein interaction data. However, some of the studies require an initial step to reduce the number of genes to be considered as differentially expressed which might be challenging for a highly heterogeneous sample set. Also the studies do not address potential inconsistencies in pathway annotations that might lead to confusion about the relevance of the findings with regard to well known cancer genes. For example, the BioCarta ERK pathway consists of 28 genes including EGFR, IGF1R, and other receptor tyrosine kinases (RTKs) while the well established cancer genes ERBB2/3/4 are not included in the pathway. In turn, ERBB2–4 and EGFR are separately annotated components of the BioCarta HER2-pathway, however, redundancies in the annotation arise from annotation of RAF, MEK, and ERK as additional HER2-pathway entities. Thus, a method that directly uses normalized expression data as well as enhanced signaling network information based on the functional relevance of a gene within a signaling network might be considered as an alternative approach to sample stratification.

To explore the possible value of such a top down network-based classification approach we here compile a comprehensive list of cancer-related signaling genes that we group into distinct signaling core modules. To distinguish the core module genes with direct signaling capacity from genes known for their rather indirect effect on core modules, we define separate stimulating or inhibiting modifier modules for those peripheral pathway components. Thereby, we establish an interconnected network of cancer relevant signaling modules mainly focusing on RTK-triggered pathways as well as the HEDGEHOG, WNT, and NOTCH pathways. Subsequently we identify expression values of individual network genes from publicly available cancer sample expression data and compute the median expression level of all module genes within a given sample as a representative measure for network module activity. For this analysis we use published data sets of breast and ovarian cancer expression profiling studies as representatives of cancer types with a high prevalence in the world population (Forouzanfar et al., 2011).

Network module activity is then used for hierarchical and *k*-means clustering to assess the networks usability for distinguishing certain subtypes of cancer samples within the larger sample cohort. We evaluate our network module-based clustering approach by comparing it with established cancer specific discriminatory gene signatures. Our results suggest that our alternative top down network approach performs well when applied to cancers of different origin.

## Results

### Compilation of comprehensive modules of cancer-related signaling pathways

For compilation of a network model of cancer-related signaling pathways we identified signaling pathway components based on information from the REACTOME pathway database (Matthews et al., 2009; Croft et al., 2011) and screening of literature using PUBMED guided by information provided by relevant review articles (Hanahan and Weinberg, 2000, 2011; Shawver et al., 2002; Perona, 2006; Lemmon and Schlessinger, 2010; Witsch et al., 2010). We focused on a core network of well described and frequently transcriptionally deregulated RTK-coupled signaling pathways including EGFR/ERBB, FGFR, PDGFR, INSR, and NGF as well as the serine/threonine kinase receptors of the TGFB/BMP family (Ornitz et al., 1996; Nakagawara, 2001; Tallquist and Kazlauskas, 2004; Krüttgen et al., 2006; Zhang et al., 2006; Benyoucef et al., 2007; Massagué, 2008; Acevedo et al., 2009; Belfiore et al., 2009; Turner and Grose, 2010; Wesche et al., 2011; Pollak, 2012; Yarden and Pines, 2012). In addition, we consider as relevant the non-kinase receptor coupled signaling pathways NOTCH, HEDGEHOG, and WNT (Jarriault et al., 1995; Veeman et al., 2003; Reya and Clevers, 2005; Klinakis et al., 2006; Palomero et al., 2006; Sharma et al., 2006; Weng et al., 2006; Polakis, 2007; Angers and Moon, 2009; Fortini, 2009; MacDonald et al., 2009; Theunissen and de Sauvage, 2009; Ranganathan et al., 2011). RTK-triggered signaling pathways use receptor specific adaptor protein complexes to couple the activated ligand/receptor upstream complexes to cytoplasmic downstream signaling cascades including RAS/MEK/ERK, JNK, p38, and PI3K/AKT signaling (Lowenstein et al., 1992; Buday et al., 1994; Birge et al., 1996; Okada and Pessin, 1996; Li et al., 2001; Ravichandran, 2001; Malumbres and Barbacid, 2003; Gotoh, 2008; Ursini-Siegel and Muller, 2008) as well as to CDC42/RAP1/RAC1, small GTPases which regulate actin dependent cell movements and also activation of JNK signaling (Feller, 2001; Eden et al., 2002; Miyamoto et al., 2004). In addition to recruitment of adaptor proteins, activated RTKs present a binding and activating interface for several other cancer relevant signaling cascades including PLCG, SRC, and STAT (Noh et al., 1995; Kamat and Carpenter, 1997; Ishizawar and Parsons, 2004; Yu et al., 2009). Our network contains distinct modules covering these RTK-specific adaptor and signaling proteins as well as the downstream cascades that transduce the growth factor signal to the level of transcriptional regulation. In the case of HEDGEHOG and WNT signaling, ligands are bound by seven-transmembrane-domain proteins (Smoothened or Frizzled class) with help of co-receptors. Signaling events are then initiated via recruitment of cytoplasmic factors. In case of NOTCH signaling, the initiation of downstream signaling events includes a series of tightly regulated cleavage events that, upon ligand binding, lead to the proteolytic release of the transcriptionally active cytoplasmic tail of NOTCH (NICD).

**Figure 1:**
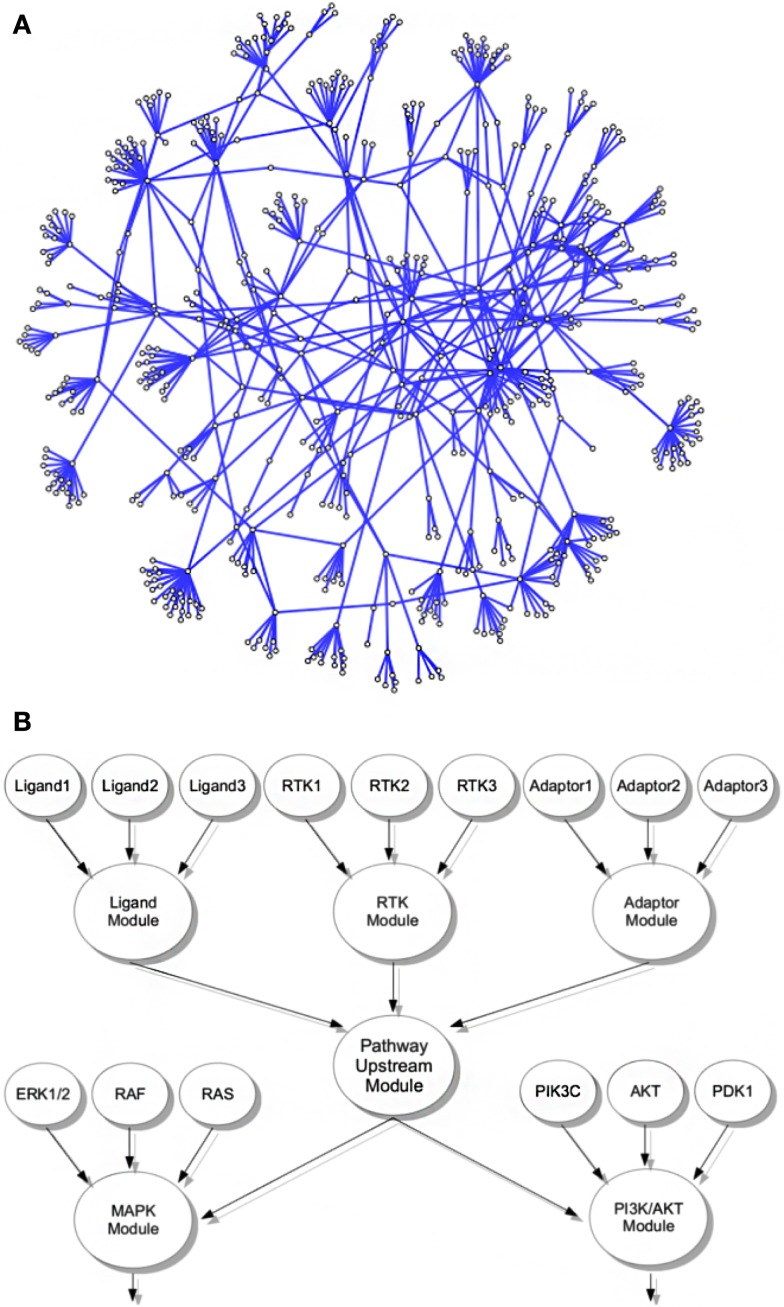
**Interconnected signaling network of cancer-related genes and modules**. **(A)** Representation of the cancer relevant signaling network including all modules and contributing genes. **(B)** Exemplified schematic layout of the module grouping strategy; all genes in the network were associated with functional modules, e.g., genes encoding ligands, RTKs, or adaptor proteins of a given pathway were grouped into the corresponding ligand, RTK, or adaptor modules. In living cells, these modules contribute to activity of the pathways upstream signaling, here represented by a pathway upstream module. Functionally, the pathway upstream module of a given pathway activates or inhibits the activity of distinct downstream modules, here exemplified for activation of a MAPK and a PI3K/AKT module. These modules themselves consist of associated genes, e.g., encoding for ERK, RAS, or RAF isoforms in case of MAPK signaling or PI3K catalytic and regulatory isoforms as well as AKT, PTEN, and others. In our network, these downstream modules are again linked to events acting further downstream in the pathway, like activation of an AP1 or MYC transcription factor module.

The principal structure of our network assumes that functionally similar core genes can be grouped into the same module within the network, exemplified by a *LIGAND-*, a *RTK/RECEPTOR-*, and an *ADAPTOR* module (Figure 1B). Factors that are themselves not bona fide ligands, RTKs, or adaptor proteins but are known to modify activity of a pathways upstream modules are grouped into separate *CoActivator* and *CoInhibitor* modules, respectively. In the network upstream modules connect to downstream modules containing the genes encoding for proteins that transduce the signal toward transcription factors and subsequent gene regulatory events. These core downstream modules are complemented by separate modules covering genes encoding for factors with indirect modulating effects on the level of signal transduction cascades. We believe that separating indirectly activating and inhibiting factors from pathway core modules allows assessment of the functional importance of a module throughout different levels of the network hierarchy more directly than in previously published pathway databases (KEGG/REACTOME) that tend to assign core components as well as components with more general modifying function to the same pathway. The nine major cancer-related signaling pathways that we assembled into a module-based network consist of 719 edges and 592 nodes covering 558 genes (Figure 1A; Table S1 in Supplementary Material).

### Application of the signaling network for analysis of pathway activity in cancer samples

Signaling activity in breast and ovarian cancer has been reported to be perturbed on the level of expression of several genes that correspond to upstream signaling modules represented in our network (Bell et al., 2011; Koboldt et al., 2012). We therefore reasoned that module-based network activity could be applied to distinguish subsets of cancer samples of these two origins. To address this point, we first mapped the overlap of all genes in our signaling network with two independent publicly available breast cancer or ovarian cancer gene-expression data sets, respectively. We then determined the network module activity for each of the samples by calculating for all modules the median expression value of all genes attributed to the same module (Anglesio et al., 2008; Lu et al., 2008; Tothill et al., 2008). We chose these particular data sets for our analysis as the sample sizes are relatively large, allowing to compute meaningful *p*-values in the statistical analysis of a *k*-means clustering approach. Also, most samples from these studies are annotated well with respect to multiple clinical markers in each of the studies, an annotation depth rarely seen in other publicly available data sets. Therefore, we reasoned that these data sets allow us to simultaneously test for the association of network module activity with several phenotypic markers at once.

**Figure 2:**
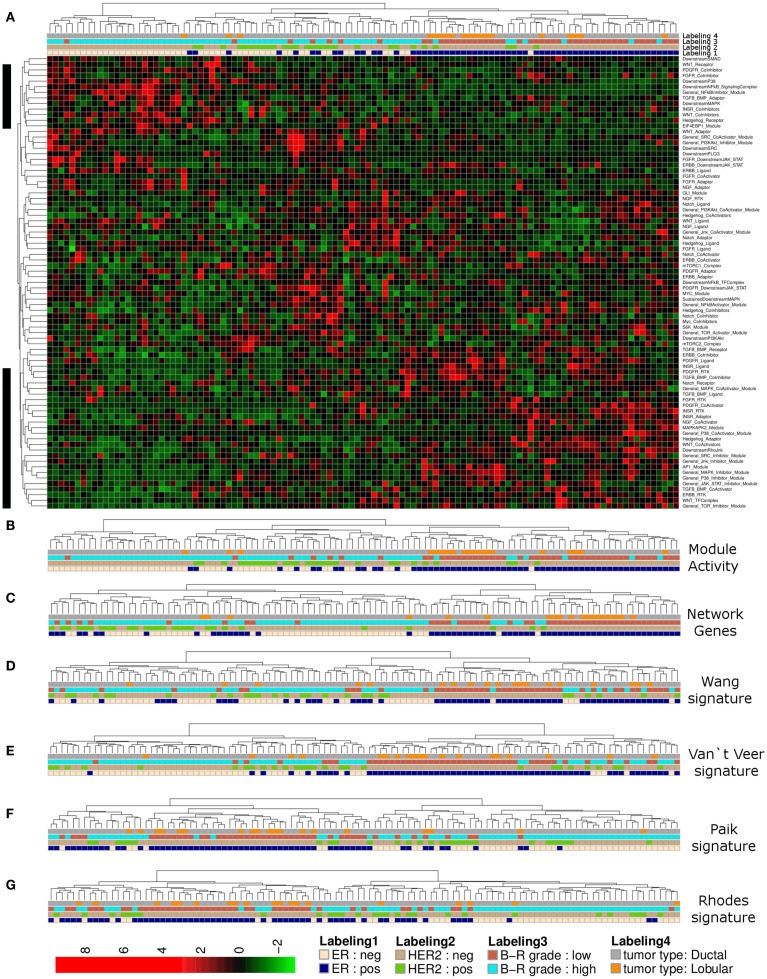
**Hierarchical clustering of a breast cancer sample set**. **(A)** Heatmap representation of a complete linkage hierarchical bi-clustering of network module activity, based on expression data of 113 breast cancer samples of Lu et al. (2008) data set; the resulting dendrogram is annotated according to the clinical sample annotation using the color code as indicated in the legend; labelings are from top to down: labeling 4, tumor type; labeling 3, tumor grade; labeling 2, HER2 expression status; labeling 1, estrogen receptor (ER) expression status; regions analyzed in more detail in Figures 3A,B are indicated by black vertical bars. **(B)** Magnification of the dendrogram from **(A)** showing the cluster of mainly ER-negative, mainly HER2-negative, high grade ductal samples that is separated from all other samples. **(C)** Cluster dendrogram resulting from bi-clustering based on expression values of all individual genes represented in the network. **(D–G)** Cluster dendrograms resulting from bi-clustering of the breast cancer samples based on expression values of published discriminative gene signature sets generated specifically for breast cancer samples **(D)** Wang et al. (2005) **(E)** van’t Veer et al. (2002) a signature generated more generally to distinguish cancer samples from each other **(F)** Rhodes et al. (2004) and another breast cancer specific signature from Paik et al. (2004) **(G)**.

Complete linkage hierarchical clustering using network module activity is consistent with two main clusters of breast cancer samples from the Lu et al. (2008) data set (Figures 2A,B). The ductal samples in the left cluster appear to be differentiated from all other samples mainly by their estrogen receptor (ER) and HER2 expression statuses and high tumor grade (Figure 2B). In comparison, clustering the expression values of all network genes results in an apparently less stringent sample discrimination regarding some of the clinical sample properties, e.g., high grade ER-negative samples co-cluster with comparably more ER-positive samples (Figure 2C). To qualitatively compare our network-based clustering with established breast cancer specific discriminative gene signatures, we performed hierarchical bi-clustering using those gene-expression values that were covered by the breast cancer primary data. We used three breast cancer specific gene signatures (van’t Veer et al., 2002; Paik et al., 2004; Wang et al., 2005) and a signature developed for discrimination of tumor grades of multiple different solid tumors (Rhodes et al., 2004). Clustering the breast cancer samples according to the van’t Veer et al. (2002) and Wang et al. (2005) gene-expression signature resulted in separation of two main clusters of similar size, where one cluster is dominated by high grade ER-negative, HER2-negative samples (Figures 2D,E). Essentially similar results are obtained using the Paik et al. (2004) and Rhodes et al. (2004) gene signatures (Figures 2F,G).

**Figure 3:**
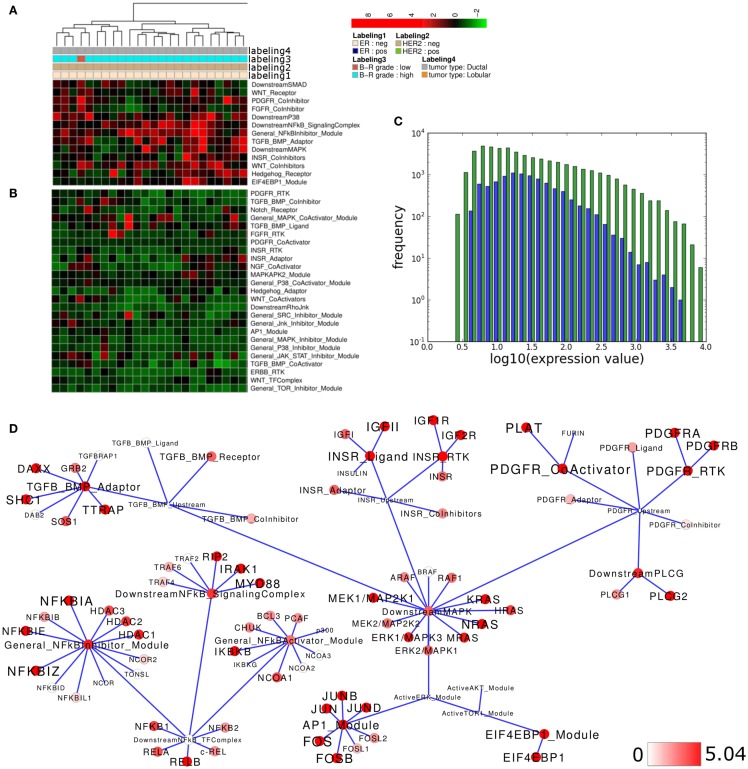
**Pathway activity analysis of a high grade breast cancer subcluster**. **(A,B)** Regions of the heatmap in Figure 2A that appear to distinguish high grade ER-negative HER2-negative samples from all other samples in the Lu et al. (2008) data set. **(C)** Plot showing distribution of log10 values of all network module activity values (blue bars) for all breast cancer samples versus log10 gene-expression values (green bars) of all genes associated with the network. **(D)** Network diagram of the top 15% differentially expressed modules comparing the difference between average network module activity in high grade versus all other samples; node color and node label size are initialized with log2 difference of the average module and gene-expression values of high grade versus all other samples, respectively; a dark red color indicates log2 difference ≥5.04 as a cutoff for the largest 15% fraction of differentially expressed network modules or genes (max. 9.28).

We noted that activity of several modules associated with the same signaling branch appear to contribute most to separation of the high grade ER-negative and HER2-negative cluster from all other samples of the data set (Figures 3A,B). Generally, the distribution curve of network module activity values is overall similar to the expression values of the genes that are associated with these modules; as one would expect from calculated median gene-expression values the network module activity value distribution is slightly more confined with regard to maximal and minimal values and shows a reduced amplitude (Figure 3C). We concluded that the overall network module activity reflects the general expression dynamics within the network. We next sought to identify modules and candidate genes attributed to those pathway modules that show differential activation and expression levels between sample subsets. To visualize the differences in average gene expression and network module activity between the high grade ER- and HER2-negative cluster and all other samples we mapped the differences to a subset of the signaling network containing the most deregulated modules (top 15% of differential module activity/gene expression; Figure 3D). Apparently, the modules/genes showing the largest difference between the clusters are related to the INSR and PDGFR pathways as well as the MAPK, NFkB, and AP1 branches. For example, the lower *PDGFR-RTK* module activity in the high grade cluster comparing to all other samples appears to result from differential expression of both PDGFRA and PDGFRB while the lower *PDGFR-CoActivator* module activity appears to result from expression differences of the PLAT/tissue Plasminogen Activator (tPA), which is, among other functions, a proteolytic activator of PDGFC (Fredriksson et al., 2004). *INSR-RTK* and *INSR-Ligand* module activity differences appear to arise primarily from reduced expression of IGF1R, IGF2R, and IGF2. These findings suggest, that PDGFR- and INSR-pathway sub-network activity in the high grade breast cancer samples of the Lu et al. (2008) data set might be relatively low compared to all other samples due to signaling modulation on the level of growth factor reception as well as on the level of the enzyme that mediates the posttranslational activation of the signaling pathway. In contrast, network module activity of the TGF/BMP-Adaptor, the Downstream-MAPK, AP1, the NFkB-SignalingComplex, and the NFkB-Inhibitor modules might be dominated in the high grade cluster relative to all other samples due to the expression differences of SHC1, MEK1, FOS-isoforms, NFkBIA/E/Z, IRAK1, and MYD88, respectively.

**Figure 4:**
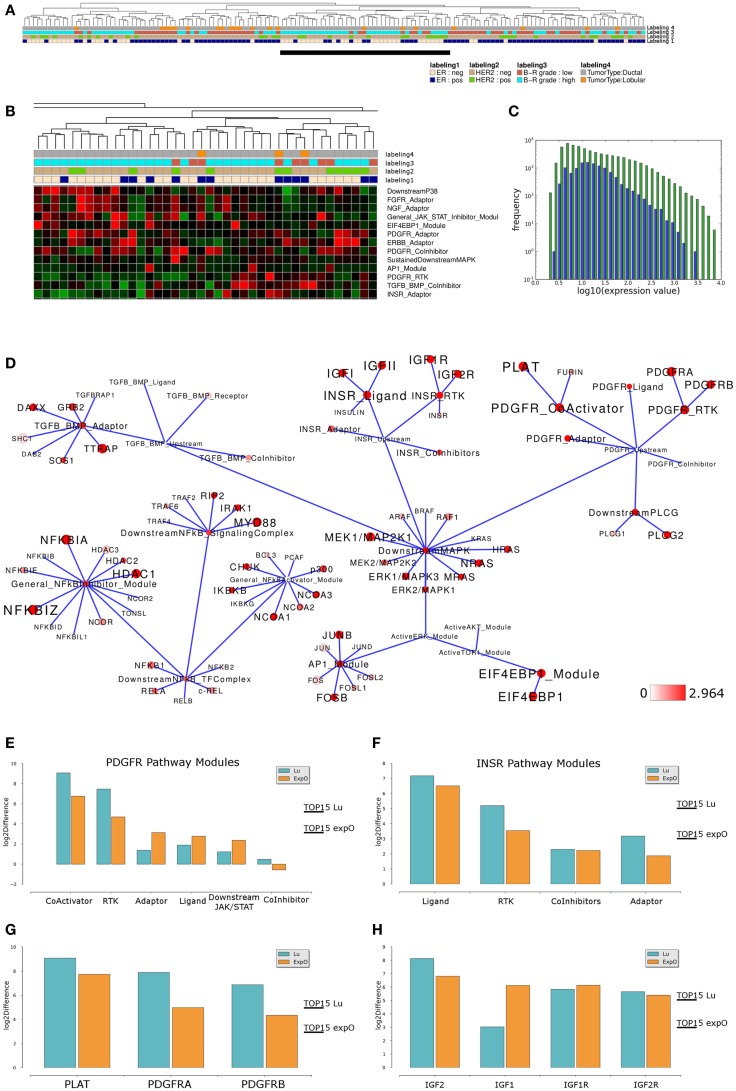
**Comparable network module activity differences in high grade breast cancer samples of an independent data set**. **(A)** Dendrogram representation of a complete linkage hierarchical bi-clustering of network module activity-based on expression data of 145 breast cancer samples of the expO data set; the resulting dendrogram is annotated according to the clinical sample annotation using the color code as indicated in the legend; labelings are from top to down: labeling 4, tumor type; labeling 3, tumor grade; labeling 2, HER2 expression status; labeling 1, estrogen receptor (ER) expression status. A cluster of mainly high grade ER and HER2-negative samples analyzed further is indicated by a black vertical bar. **(B)** Magnification of module activities that appear to distinguish the cluster of high grade ER and HER2-negative samples from all other samples within the expO data set. **(C)** Plot showing distribution of log10 values of all network module activity values (blue bars) for all expO breast cancer samples versus log10 distribution of gene-expression values (green bars) of all genes associated with the network. **(D)** Network diagram of the top 15% differentially expressed modules comparing the difference between average network module activity in high grade versus all other samples; node color and node label size are initialized with log2 difference of the average module and gene-expression values of high grade versus all other samples; red color indicates expression above a log2 difference ≥2.964 (max. 7.742). **(E,F)** Comparison of log2 differences in **(E)** average PDGFR and **(F)** average INSR-pathway modules activity as calculated for the Lu (blue bars) and expO (orange bars) data sets. The thresholds for determination of the top 15% differentially activated modules within both data sets are indicated by the horizontal bars. **(G)** Comparison of log2 differences in average *PDGFR* module related gene-expression values as calculated for the Lu and expO data sets. **(H)** Comparison of log2 differences in average *INSR* module related gene-expression values as calculated for the Lu and expO data sets. For comparison of differences of individual gene expression with the differences in module activities, the thresholds for determination of the top 15% differentially activated modules within both data sets are indicated by the horizontal bars.

We next asked if similar pathway activity in high grade ER-, HER2-negative breast cancer samples could be identified in an independent data set. To address this point, we performed an analogous analysis of breast cancer samples provided by the Expression Project for Oncology data set (expO; expo.intgen.org/geo/home.do). Clustering the network module activity of these samples results in separation of one main-cluster containing a mixed population of high and low grade, ER-negative or positive but predominantly HER2-negative samples from all other samples constituting the heterogeneous second main cluster on the right hand side (Figure 4A). This second main-cluster comprises a large subcluster of mainly high grade ER- and HER2-negative samples and we chose this subcluster for further analysis of differential module activity comparing to all other samples. Modules contributing to sample clustering were identified (Figure 4B) and generally the distribution of network module activity again reflects the distribution of gene-expression associated with the network (Figure 4C). We then mapped the differentially activated modules/genes to the relevant sub-network as described above and we find that the network of differential module and gene activity from this second set of breast cancer samples partially overlaps with the sub-network identified as relevant for the Lu et al. (2008) data set. In particular, modules related to the PDGFR and INSR related subnetworks score among the top 15% of differentially activated modules and both pathways thereby show highly similar differential activity between high grade and all other samples in both data sets (Figure 4D). Comparing module activity values for both data sets shows that *PDGFR-CoActivator* and *PDGFR-RTK* modules can be identified as differentially activated above threshold in both data sets while *PDGFR-Adaptor*, *PDGFR-Ligand PDGFR-CoInhibitor*, and *PDGFR-Downstream-JAK/STAT* modules do not show differential activation in both data sets (Figure 4E). Similarly, the *INSR-Ligand* and *INSR-RTK* modules show differential activation above threshold in both data sets while *INSR-CoInhibitor* and *INSR-Adaptor* modules are not identified as differentially activated (Figure 4F). We conclude that a subset of PDGFR and INSR-pathway modules scores among the top 15% differentially activated modules as judged by thresholds generated for each of the data sets individually suggesting that those modules are differentially activated between high grade and all other samples in a way that is comparable between the data sets. When we compared differential expression of those genes that appear to contribute to the differential network module activity we found that for the PDGFR signaling branch the differential expression between high grade and all other samples of PDGFRA, PDGFRB, and PLAT exceeds the threshold in both data sets (Figure 4G). For the INSR branch we find that the IGF2 ligand as well as the IGF1R RTK and IGF2R show similar differences between the high grade cluster and all other samples in both data sets while the IGF1 ligand appears to be differentially expressed in the expO data set only (Figure 4H). Taken together, our analysis suggests that our network-based approach might be feasible for hierarchical clustering to identify interesting sub-populations in breast cancer data sets and for a first visual inspection of expression differences in these data sets preceding a more detailed analysis and verification of the underlying gene regulatory changes.

### Analysis of pathway activity of ovarian cancer samples

**Figure 5:**
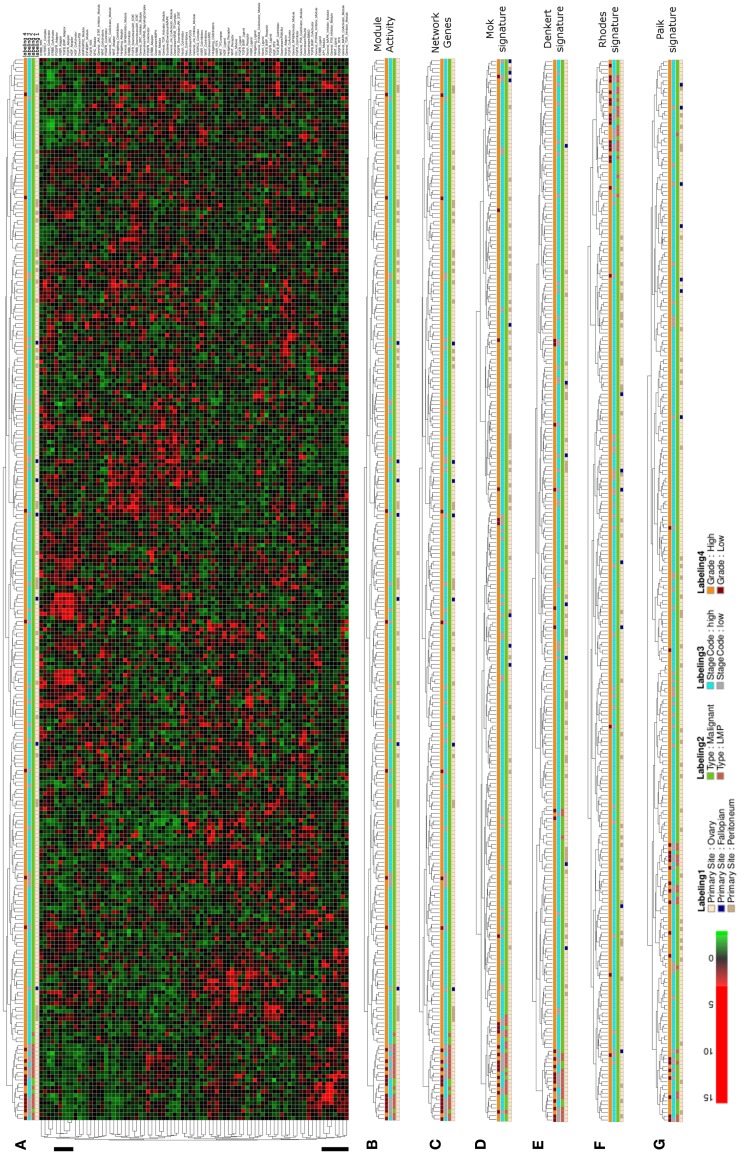
**Hierarchical clustering of an ovarian cancer sample data set**. **(A)** Heatmap representation of a complete linkage hierarchical bi-clustering of network module activity, based on expression data of 278 ovarian cancer samples derived from the Tothill et al. (2008) data set; the resulting sample distribution dendrogram is annotated according to the clinical sample annotation using the color code as indicated in the legend; labelings are from top to down: labeling 4, consolidated tumor grade; labeling 3, stage code low (I and II) or high (III and IV); labeling 2, cancer type LMP or malignant; labeling 1, primary tumor site ovary, fallopian tube, or peritoneum. Regions analyzed in more detail in Figure 6A are indicated by black vertical bars. **(B)** Magnification of the dendrogram from **(A)**. **(C)** Cluster dendrogram resulting from bi-clustering based on expression values of all individual genes represented in the network. **(D–G)** Cluster dendrograms resulting from bi-clustering of the samples based on expression values of published discriminative gene signature sets generated specifically for ovarian cancer samples **(D)** Mok et al. (2009), **(E)** Denkert et al. (2009), as well as the signatures by **(F)** Rhodes et al. (2004), and **(G)** Paik et al. (2004).

In the next step of our analysis we wanted to test whether our network-based stratification approach might also be applicable to another cancer type and we therefore performed hierarchical clustering of ovarian cancer samples of a large patient cohort that is well annotated with regard to clinical parameters as provided by Tothill et al. (2008). Hierarchical clustering of network module activity of these samples results in separation of two main clusters, where the left main-cluster contains one sub-cluster enriched for low malignant potential (LMP), low grade and low stage samples of ovarian origin (Figures 5A,B). The other samples in the left main cluster as well as the second main-cluster contain mainly malignant, high stage and high grade samples. Co-clustering of LMP samples into one distinct subcluster is as well achieved using the network gene expression for clustering (Figure 5C). To compare network module activity clustering with established signature approaches, we performed clustering of samples using two different ovarian cancer specific gene signatures (Denkert et al., 2009; Mok et al., 2009), the general discriminatory signature by Rhodes et al. (2004) and the breast cancer specific signature described by Paik et al. (2004). Using the Mok et al. (2009) signature results in a sample distribution between the main clusters that is very similar to network module activity and network gene-expression values, including separation of all LMP samples into one subcluster (Figure 5D). Clustering of the Denkert et al. (2009), Rhodes et al. (2004), and Paik et al. (2004) gene signatures results in less stringent separation of LMP samples from all other samples (Figures 5E–G). Notably, the breast cancer specific signature described by Paik et al. (2004) shows the least efficient co-clustering with regard to the LMP samples comparing to all other signatures (Figure 5G).

**Figure 6:**
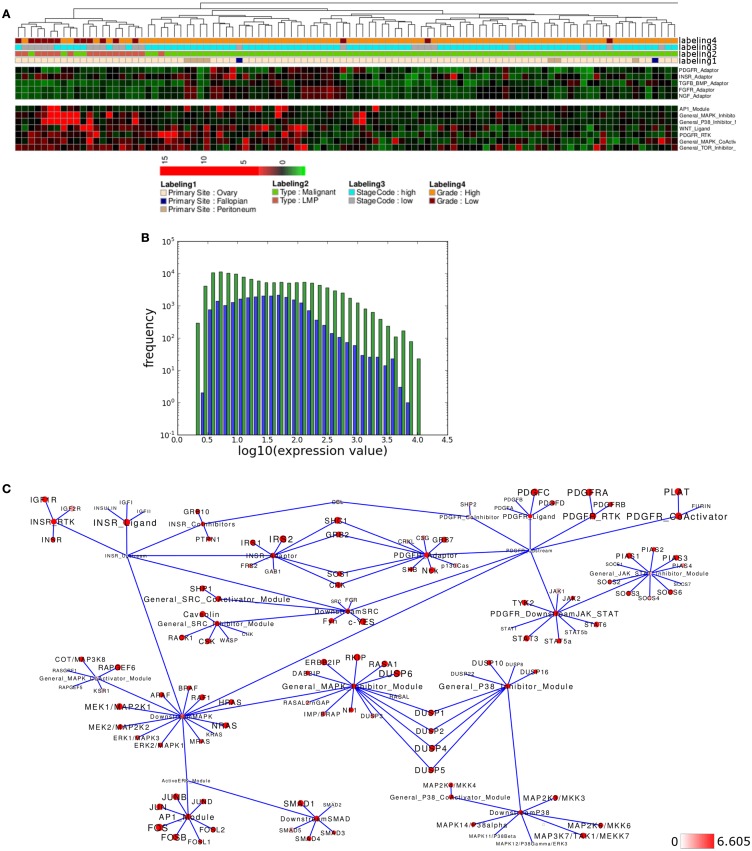
**Pathway activity analysis of the LMP ovarian cancer subcluster**. **(A)** Regions of the heatmap in Figure 5 that appear to distinguish low malignancy potential (LMP) samples from all other ovarian cancer samples. **(B)** Plot showing distribution of log10 values of all network module activity values (blue bars) for all Tothill et al. (2008) ovarian cancer samples versus log10 distribution of gene-expression values (green bars) of all genes associated with the network. **(C)** Network diagram of the top 15% differential expressed modules and genes comparing the difference between average network module activity in LMP versus all other samples; node color and node label size are initialized with log2 difference of the average gene-expression values of LMP versus all other samples; red color indicates expression above a log2 difference ≥6.605 (max. 9.768).

Several network modules appear to show differential activity in the LMP- and co-clustering high grade samples versus all other samples (Figure 6A). Again module activity in the network appears to reflect the overall distribution of the expression of all network associated genes (Figure 6B). To look into details of network module activity differences between LMP and all other samples, we again calculated the average expression levels for both groups separately and then mapped the top 15% of differentially activated modules to the relevant signaling subnetwork (Figure 6C). Interestingly, *INSR-RTK*, *PDGFR-CoActivator*, and *PDGF-RTK* modules as well as the *AP1* and *MAPK-Inhibitor* modules were identified among the top differentially activated modules. Here, *INSR-RTK*, *AP1*, and *MAPK-Inhibitor* module activities appears to be higher, while *PDGFR-RTK* and *PDGFR-CoActivator* module activities as well as *PDGFR-* and *INSR-ADAPTOR* modules appear lower activated in LMP versus all other samples.

**Figure 7:**
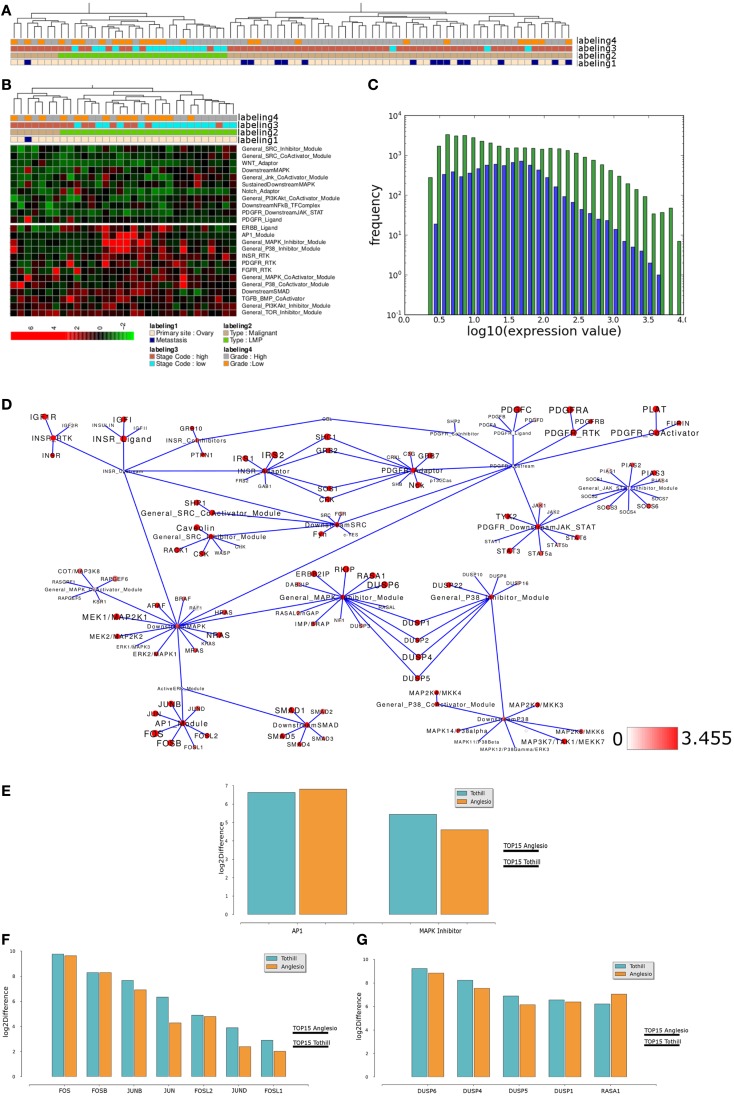
**Comparable network module activity differences between LMP and all other ovarian cancer samples of an independent data set**. **(A)** Dendrogram representation of a complete linkage hierarchical bi-clustering of network module activity, based on expression data of 83 ovarian cancer samples of the Anglesio et al. (2008) data set; the resulting dendrogram is annotated according to the clinical sample annotation using the color code as indicated in the legend; labelings are from top to down: labeling 4, consolidated tumor grade; labeling 3, stage code low (I and II) or high (III and IV); labeling 2, cancer type LMP or malignant; labeling 1, primary tumor site ovary or metastasis. **(B)** Depiction of network module activities that appear to distinguish the cluster of LMP samples from all other samples within the Anglesio et al. (2008) data set. **(C)** Plot showing distribution of log10 values of all network module activity values (blue bars) for all Anglesio et al., ovarian cancer samples versus log10 distribution of gene-expression values (green bars) of all genes associated with the network. **(D)** Network diagram of the top 15% differentially expressed modules comparing the difference between average network module activity in LMP versus all other samples; node color and node label size are initialized with log2 difference of the average module activity and gene-expression values of LMP versus all other samples; dark red color indicates expression above a log2 difference ≥3.455 (max. 9.631). **(E)** Comparison of log2 differences in average *AP1* and *MAPK-Inhibitor* module activity as calculated for the Tothill (blue bars) and Anglesio (orange bars) data sets. **(F)** Comparison of log2 differences in average *AP1* module associated gene-expression values as calculated for the Anglesio et al. (2008) and Tothill et al. (2008) data sets. **(G)** Comparison of log2 differences in average *MAPK-Inhibitor* module associated gene-expression values as calculated for the Anglesio et al. and Tothill et al. data sets. For comparison of differences of individual gene expression with the differences in module activities, the thresholds for determination of the top 15% differentially activated modules within both data sets are indicated by the horizontal bars.

To test if similar differences in network module activity might be identified in an independent data set, we performed analogous analysis with samples provided by Anglesio et al. (2008) that also include LMP samples. Using network module activity for hierarchical clustering results in two main clusters, one containing all LMP samples while the other cluster contains only malignant and all but one of the metastasis samples (Figure 7A). Again, activity of several modules appears to distinguish the LMP cluster from all other samples (Figure 7B) and, for example, the *INSR-RTK*, *PDGFR-CoActivator*, and *PDGF-RTK* modules as well as the *AP1*, *MAPK-Inhibitor* modules show differential activity between LMP and all others samples, a finding that is highly similar to the situation found in the Tothill et al. (2008) data set. We again tested if module activity in the network appears to reflect the overall distribution of the expression of all network associated genes, and this appears to be the case (Figure 7C). Mapping of the top 15% differences of network module activity and average gene expression between both sample groups to the relevant signaling subnetwork results in a network activity pattern comparable between Tothill and Anglesio data sets (Figure 7D). Indeed, direct comparison of, e.g., *AP1* and *MAPK-Inhibitor* differential module activity shows that both modules are similarly differentially activated between LMP and all other samples in both data sets (Figure 7E). We then evaluated the contribution of differential gene expression to the observed differences in *AP1* and *MAPK-Inhibitor* module activity and find that in the case of the *AP1* module five of seven genes are similarly differentially expressed between LMP and all other samples in both data sets, namely FOS, FOSB, FOSL2 as well as the JUN and JUNB genes (Figure 7F). In case of the *MAPK-Inhibitor* the differential expression of 5 of 20 genes appears to contribute most to the observed differential module activity, namely the expression of DUSP1, DUSP4, DUSP5, DUSP6, and RASA1, that are similarly differentially expressed comparing LMP and all other samples in both data sets (Figure 7G). Taken together, our results with breast and ovarian cancer sample clustering suggest that our top down network module activity-based approach is feasible for sample stratification and initial follow up analysis for two different types of cancers of different origins.

As hierarchical clustering provides a tool for visualization of sample distribution between clusters but provides no quantitative measure for clustering success we wanted to further substantiate the assumption that our module-based network approach is applicable for sample stratification using *k*-means clustering as an independent, unsupervised method. We determined the optimal number of expected clusters as two, using the clValid tool for both the breast and ovarian cancer sample sets. As most samples in the data set are well annotated with respect to clinically determined parameters, we analyzed those sample’s distribution by the *k*-means algorithm regarding to known phenotypic representations. We then calculated a *p*-value for each of the clustering results using a chi-square test which can be used as a measure to compare clustering success of different signatures to our network module activity approach. For both the breast cancer Table 1 and ovarian cancer Table 2 data sets we find that *k*-means clustering using network activity results in separation of samples into the expected number of clusters equally well as clustering using the well established gene signatures for ovarian and breast cancer, as judged by similarity of *p*-values achieved. Thus, *k*-means clustering provides further evidence that network module activity might be a valid method for sample clustering, at least for some of the selected parameters like ER grade in case of breast cancer and stage code in case of the ovarian cancer samples. Our results suggest that network module activity might indeed be a useful alternative method for clustering of cancer samples of different origins and performs well comparing to established discriminatory gene sets.

**Table 1:** ***k*-Means clustering of breast cancer samples**.

Lu et al. dataset				expO dataset			

	
Network activity values	cluster 1	cluster 2	*p*-value	Network activity values	cluster 1	cluster 2	*p*-value
ER:neg	19	33	6.14E-04	ER:neg	29	24	5.31E-01
ER:pos	43	18		ER:pos	44	48	
HER2:neg	46	39	9.52E-01	HER2:neg	53	54	8.89E-01
HER2:pos	16	12		HER2:pos	20	18	
B-R grade:low	28	18	3.84E-01	B-R grade:low	30	35	4.58E-01
B-R grade:high	34	33		B-R grade:high	43	37	

**Oncotype signature**	**cluster 1**	**cluster 2**	***p*-value**	**Oncotype signature**	**cluster 1**	**cluster 2**	***p*-value**

ER:neg	17	35	2.66E-04	ER:neg	29	24	9.97E-01
ER:pos	42	19		ER:pos	49	43	
HER2:neg	45	40	9.58E-01	HER2:neg	55	52	4.36E-01
HER2:pos	14	14		HER2:pos	23	15	
B-R grade:low	28	18	1.82E-01	B-R grade:low	33	32	6.24E-01
B-R grade:high	31	36		B-R grade:high	45	35	

**Rhodes et al. signature**	**cluster 1**	**cluster 2**	***p*-value**	**Rhodes et al. signature**	**cluster 1**	**cluster 2**	***p*-value**

ER:neg	18	34	2.90E-04	ER:neg	24	29	8.69E-01
ER:pos	43	18		ER:pos	39	53	
HER2:neg	45	40	8.66E-01	HER2:neg	43	64	2.55E-01
HER2:pos	16	12		HER2:pos	20	18	
B-R grade:low	25	21	8.99E-01	B-R grade:low	20	45	9.11E-03
B-R grade:high	36	31		B-R grade:high	43	37	

**Network genes**	**cluster 1**	**cluster 2**	***p*-value**	**Network genes**	**cluster 1**	**cluster 2**	***p*-value**

ER:neg	37	15	1.06E-04	ER:neg	23	30	3.98E-01
ER:pos	20	41		ER:pos	48	44	
HER2:neg	43	42	8.70E-01	HER2:neg	53	54	9.68E-01
HER2:pos	14	14		HER2:pos	18	20	
B-R grade:low	21	25	5.14E-01	B-R grade:low	35	30	3.72E-01
B-R grade:high	36	31		B-R grade:high	36	44	

**van’t Veer et al. signature**	**cluster 1**	**cluster 2**	***p*-value**	**van’t Veer et al. signature**	**cluster 1**	**cluster 2**	***p*-value**

ER:neg	36	16	2.40E-04	ER:neg	23	30	9.43E-01
ER:pos	20	41		ER:pos	38	54	
HER2:neg	43	42	8.70E-01	HER2:neg	46	61	8.52E-01
HER2:pos	13	15		HER2:pos	15	23	
B-R grade:low	22	24	9.10E-01	B-R grade:low	31	34	2.86E-01
B-R grade:high	34	33		B-R grade:high	30	50	

**Wang et al. signature**	**cluster 1**	**cluster 2**	***p*-value**	**Wang et al. signature**	**cluster 1**	**cluster 2**	***p*-value**

ER:neg	37	15	2.38E-05	ER:neg	24	29	7.08E-01
ER:pos	18	43		ER:pos	46	46	
HER2:neg	41	44	9.55E-01	HER2:neg	53	54	7.50E-01
HER2:pos	14	14		HER2:pos	17	21	
B-R grade:low	19	27	2.68E-01	B-R grade:low	41	24	2.30E-03
B-R grade:high	36	31		B-R grade:high	29	51	

Results of *k*-means-based clustering of the network module activity or expression values for all network genes from the Lu et al. (2008) and expO data sets in comparison to the clustering results based on the expression value of genes overlapping the signature gene sets of Wang et al. (2005), van’t Veer et al. (2002), Paik et al. (2004) or Rhodes et al. (2004).

**Table 2:** ***k*-Means clustering of ovarian cancer samples**.

Tothill et al. dataset				Anglesio et al. dataset			

	
Network activity values	cluster 1	cluster 2	*p*-value	Network activity values	cluster 1	cluster 2	*p*-value
Type:malignant	117	144	1.80E-01	Type:malignant	28	30	2.81E-01
Type:LMP	11	6		Type:LMP	16	9	
Stage code:high	100	137	3.43E-03	Stage code:high	34	30	8.23E-01
Stage code:low	28	13		Stage code:low	10	9	
Grade:high	118	141	7.20E-01	Grade:high	29	29	5.50E-01
Grade:low	10	9		Grade:low	15	10	

**Denkert et al. signature**	**cluster 1**	**cluster 2**	***p*-value**	**Denkert et al. signature**	**cluster 1**	**cluster 2**	***p*-value**

Type:malignant	125	136	5.33E-01	Type:malignant	33	25	2.40E-01
Type:LMP	10	7		Type:LMP	10	15	
Stage code:high	106	131	3.65E-03	Stage code:high	35	29	4.82E-01
Stage code:low	29	12		Stage code:low	8	11	
Grade:high	127	132	7.30E-01	Grade:high	33	25	2.40E-01
Grade:low	8	11		Grade:low	10	15	

**Mok et al. signature**	**cluster 1**	**cluster 2**	***p*-value**	**Mok et al. signature**	**cluster 1**	**cluster 2**	***p*-value**

Type:malignant	107	154	8.10E-01	Type:malignant	34	24	7.51E-01
Type:LMP	7	10		Type:LMP	13	12	
Stage code:high	100	137	4.26E-01	Stage code:high	35	29	6.96E-01
Stage code:low	14	27		Stage code:low	12	7	
Grade:high	106	153	8.88E-01	Grade:high	34	24	7.51E-01
Grade:low	8	11		Grade:low	13	12	

**Oncotype signature**	**cluster 1**	**cluster 2**	***p*-value**	**Oncotype signature**	**cluster 1**	**cluster 2**	***p*-value**

Type:malignant	122	139	8.45E-01	Type:malignant	25	33	8.68E-01
Type:LMP	7	10		Type:LMP	11	14	
Stage code:high	113	124	3.92E-01	Stage code:high	29	35	6.96E-01
Stage code:low	16	25		Stage code:low	7	12	
Grade:high	120	139	8.80E-01	Grade:high	27	31	5.17E-01
Grade:low	9	10		Grade:low	9	16	

**Rhodes et al. signature**	**cluster 1**	**cluster 2**	***p*-value**	**Rhodes et al. signature**	**cluster 1**	**cluster 2**	***p*-value**

Type:malignant	125	136	4.20E-02	Type:malignant	28	30	6.84E-01
Type:LMP	13	4		Type:LMP	14	11	
Stage code:high	109	128	5.85E-03	Stage code:high	31	33	6.44E-01
Stage code:low	29	12		Stage code:low	11	8	
Grade:high	128	131	9.74E-01	Grade:high	27	31	3.76E-01
Grade:low	10	9		Grade:low	15	10	

**Network genes**	**cluster 1**	**cluster 2**	***p*-value**	**Network genes**	**cluster 1**	**cluster 2**	***p*-value**

Type:malignant	134	127	4.13E-01	Type:malignant	32	26	4.87E-01
Type:LMP	11	6		Type:LMP	11	14	
Stage code:high	116	121	1.60E-02	Stage code:high	35	29	4.82E-01
Stage code:low	29	12		Stage code:low	8	11	
Grade:high	134	125	7.79E-01	Grade:high	31	27	8.29E-01
Grade:low	11	8		Grade:low	12	13	

Results of *k*-means-based clustering of the network module activity or expression values for all network genes from the Tothill et al. (2008) and Anglesio et al. (2008) data sets in comparison to the clustering results based on the expression value of genes overlapping the signature gene sets of Mok et al. (2009), Denkert et al. (2009), Rhodes et al. (2004), and Paik et al. (2004).

## Discussion

The approach to classify cancer samples according to their gene-expression profile has become a standard method in recent years, partially driven by the hope that discriminatory gene signatures might guide treatment options for individual patients. Thus, the search for best performing discriminatory gene-expression signatures potentially covering more than one cancer type remains a relevant topic in cancer research. Generally, all methods, including ours, that rely on signaling pathway associated gene-expression levels to compare biological samples share an assumption about the most simple model hypothesis. This common model hypothesis assumes that any differential effect on a pathways mRNA expression level in a given sample is a linear functional response to changes in signaling activity followed by differential transcription of a specific subsets of mRNAs. However, this assumption might be partially confounded by changes of mRNA levels due to other effects, for example the differential activity of mRNA degradation mechanisms between the samples. The overall mRNA degradation rate in a cell depends on a complex interplay of multiple mechanisms relying on distinct protein–protein and protein-mRNA interactions (Garneau et al., 2007). It is therefore possible that differences in the expression or activity levels of any of these mRNA degradation mechanisms in cancer cells obstruct the correct identification of pathway deregulation due to effects on mRNA stability and overall mRNA abundance not appreciated by the initial hypothesis (López de Silanes et al., 2007). The identification of pathway deregulation based on mRNA expression levels could also be complicated by expression of cancer-related mRNAs containing shorter 3′ untranslated regions that result in an increased mRNA stability and more efficient translation into proteins (Mayr and Bartel, 2009). While classical expression profiling studies of cancer samples using chip hybridization might not address the expression status of 3′ UTR variants, the application of advanced methods for differential mRNA expression determination like RNA sequencing should allow appreciation of any such 3′ related effects. Furthermore, the mechanisms affecting mRNA degradation as mentioned above are either already known or highly likely to be regulated posttranslationally in the context of the proliferating cancer cell; for example protein phosphorylation downstream of ERK, JNK, or p38 signaling is relevant for regulation of mRNA decay while the molecular details of that regulation mechanism are not entirely analyzed yet (López de Silanes et al., 2007). Non-RTK-coupled signaling pathways have also been shown to affect mRNA decay in cancer cells such as the regulation of mRNA degradation in response to WNT signaling a mechanism that is under influence of signaling crosstalk by the PI3K/AKT pathway (Gherzi et al., 2006; Noubissi et al., 2006; Benjamin and Moroni, 2007). Thus, any effect of an altered mRNA degradation machinery might affect abundance of a specific subset of mRNAs under certain cellular conditions where distinct combinations of signaling pathways are activated. Recent studies began to analyze in detail the relation between mRNA transcription and degradation in response to extracellular stimuli but further work is clearly needed to extend such studies to other than the analyzed cell types and signaling events (Cheadle et al., 2005; Hao and Baltimore, 2009; Rabani et al., 2011; Pai et al., 2012). With respect to the genes associated with the signaling network presented in this study we find that some network genes are indeed known to be regulated on the level of mRNA degradation, such as the VEGF and FGF8 mRNAs (Alonso, 2012). Furthermore, preliminary bioinformatic analysis suggests that the mRNA of 17 of our network genes might contain AU-rich elements in the untranslated region (data not shown). Of these genes only DUSP1 has been identified as a relevant differentially activated gene in this study. The predicted ARE motif within the 17 genes might therefore allow regulation of mRNA stability by RNA binding proteins and degradation related effects might affect the activity of the network modules that these genes are attributed to. However, given the incomplete knowledge about altered mRNA degradation mechanisms in the analyzed samples we decided to retain the most simple model assumption for our study, namely that mRNA levels directly reflect the activity of the signaling network. A systematic analysis of the response of mRNA transcription and degradation over time in response to distinct signaling events and combinations thereof would clearly be highly informative to better understand the dynamic details of the systems biology of decay pathways in cancer samples.

In this work, we explore the idea of a top down approach for sample stratification based on a module-based network of cancer relevant signaling pathways and the approach using the network module activity as a proxy for pathway activity is consistent with previous work (Chuang et al., 2007). For quantitative comparison of our network module-based approach with published gene signatures we chose the widely used *k*-means clustering algorithm as one of the most simple and effective approaches to sample clustering (Gibbons and Roth, 2002). Using the *k*-means algorithm we tested the non-linear Spearman correlation (data not shown) and the linear Pearson correlation measures for sample clustering. The Pearson correlation measure lead to a significant sample clustering in case of ER-grade for breast cancer and tumor stage for ovarian cancer, respectively. Currently, we cannot exclude that alternative unsupervised clustering algorithms or correlation measures might lead to more significant findings for additional clinical parameters in the analyzed data sets and further work is needed to test this hypothesis. Furthermore, for assembly of our network, we considered several of the most established cancer pathways, while leaving out other signaling pathways even if they might be of relevance for a distinct group of cancer types or their general contribution to cancer development has yet to be established more firmly. Also, our network, in its current state, does not consider other cellular processes coupled to signaling events such as the DNA damage response or the core cell cycle regulating machinery which are known to be frequently deregulated in cancer (Bartek et al., 2007; Harper and Elledge, 2007; Williams and Stoeber, 2007; Basu et al., 2012). Albeit those limitations, our results suggest that the established module-based core signaling network can be useful to discriminate relevant subclusters of samples from two different cancer types. In particular, visualizing the network module activity in a heatmap-like representation of hierarchical clustering allows an immediate impression of the levels at which transcriptional deregulation takes place in the network hierarchy and might guide toward potentially interesting sub-modules for follow up analysis as we have shown for several examples in the breast and ovarian cancer data sets. In the case of the ovarian cancer samples, the identification of a potentially higher activity of the MAPK-Inhibitory module in LMP samples is consistent with previous findings. Indeed, the work of Anglesio et al. (2008) that generated one of the data sets we used for our analysis identified the expression of DUSP family members 1/4/5/6 as discriminatory for the LMP subcluster. However, the relevance of the other identified modules for the LMP sub-cluster phenotype is less clear. For example, relevance of elevated AP1 module activity for establishment or maintenance of the LMP phenotype remains to be shown by independent experiments, for example using RNAi mediated mRNA knockdown in LMP derived cell lines tested for their malignant and/or invasive potential *in vitro*. For a subset of breast cancer samples our approach indicates a potentially common relatively low activity of PDGFR- and INSR-pathways occurring at different levels within the network hierarchy. This observation in two independent data sets is consistent with the notion that a high grade ER- and HER2-negative sub-cluster might comprise primarily of samples with low aggressiveness as previous reports show that PDGFR expression is rather associated with high histopathological grade in ER-negative, HER2-positive breast cancer samples (Carvalho et al., 2005; Paulsson et al., 2009; Ahmad et al., 2011) and that INSR up-regulation is imminent in approximately 90% of breast cancer samples with bad prognosis (Nielsen et al., 2004; Law et al., 2008).

Interestingly, we find that network module activity-based clustering performs well in cancer types of two different origins comparing to established cancer specific gene signatures that have been generated specifically for each of the tumor types separately. This might be intriguing as the overlap between the genes in our network and the established discriminatory gene sets is minimal. Indeed, we identify a minimal overlap of four different network genes with the breast cancer signatures of Wang et al. (2005) (HDAC1, PIAS3) and van’t Veer et al. (2002) (DUSP4, DVL3) and a maximal overlap of 11 network genes with the ovarian cancer specific gene signature of Mok et al. (2009) (MAPK9, ARAF, PEPB1, MAPK1, TGFB3, ERBB2, ADAM12, GREM1, SMAD2, JAG2, GLI3). However, success of our network approach for sample clustering might be partially explained by the observation that several gene sets can be derived from the same set of cancer samples that perform equally well for sample stratification by clustering (Ein-Dor et al., 2005; Fan et al., 2006; Abba et al., 2010). Thus, given that the experimental outline, the underlying statistical method for gene set definition and other factors have great influence on gene signature determination it might be less surprising that our network-based gene set provides another effective means for sample discrimination. However, we believe that the discrimination of different sub-modules guided by the functional relevance of the associated genes provides a benefit over other methods using more general pathway annotations and allow assessment of pathway related gene-expression independent of prior knowledge of cancer specific effects.

It should be emphasized that the success of statistical methods to derive gene signatures for a specific cancer subtype can partially be confounded by the intrinsic heterogeneous nature of the analyzed cancer sample, e.g., with respect to altered mRNA decay related mechanisms as described above. Also, cancer samples of different origin can differ widely with respect to the mutational status of oncogenes and tumor suppressor genes and/or gene and chromosome copy number alterations, respectively. Even subtypes of one cancer type can be discriminated by mutational status as exemplified for the group of so called triple negative breast cancer. While this cancer subtype is characterized by absence of estrogen and progesterone receptors as well as HER2 over-expression, triple negative breast cancer samples appear to constitute a heterogeneous group as mutations in TP53, PIK3CA, and PTEN have recently been shown to confer cancer driving properties in most but not all cases (Curtis et al., 2012; Shah et al., 2012). Acknowledging the mutational status within a given cancer sample might therefore dramatically improve the value of any predictive method including our network module-based approach. Development of such a detailed signaling network of cancer relevant pathways in the future is therefore of great scientific and clinical interest.

## Materials and Methods

### Establishment of cancer relevant signaling module-based network

We generated a comprehensive list of human cancer-related signaling components based upon pathway information obtained from REACTOME database version 40 (Croft et al., 2011), complemented with interaction data for individual signaling adaptors, receptors/ligands, and peripheral modifiers derived from a PUBMED gene entry search. Genes encoding for ligands, receptors or RTKs as well as adaptor proteins of the same signaling pathway were then grouped into distinct modules that contribute to a common upstream module. The upstream modules were linked to pathway specific downstream modules including MAPK-, PI3K/AKT-, PLCG-, Rho/Jnk-, SRC-, JAK/STAT-, p38-, NFkB-SignalingComplex-, and SMAD-modules. Those downstream modules are connected via a pathway’s “effectors” entities (*activeERK*, *activeAKT*, etc.) to downstream transcription factor modules including MYC, AP1, GLI, and NICD transcription factors. To compare the composition of ERBB and MAPK pathway modules described in our network to previous pathway annotations we consulted the KEGG (Ogata et al., 1999), BioCarta (http://www.biocarta.com), and MSigDB databases (Subramanian et al., 2005). To identify AU-rich elements in the UTR of signaling network associated genes we used the online tool ARED 3.0 (Bakheet et al., 2006).

### Assessment of network module activity

For initialization of our network model with expression data we used raw data of two comprehensive gene-expression profiling studies each for breast and ovarian cancer that used the Affymetrix U133A or U133 plus 2.0 platforms for data generation (breast cancer GSE5460 and GSE2109, Ovarian cancer GSE9891 and GSE12172). We normalized the individual raw data using the quantile normalization method. We selected only those samples for analysis that are annotated with respect to all individual clinical markers. We then extracted the normalized expression values of all genes contributing to the network modules from those data for further analysis. We assume that a similar cellular phenotype and clinical representation of the analyzed samples might arise not only from deregulation of the exact same gene within a module at the same hierarchical step within the network but also from: (i) deregulation of normally co-expressed and functionally interchangeable proteins within the same network module and/or (ii) deregulation of the same pathway on different levels within the network hierarchy potentially involving CoActivators/CoInhibitor regulatory modules. After determining gene-expression values for all the genes of all modules a “network module activity” was calculated for each module as the median value of all the gene-expression values of the given module. We then tested the feasibility of using these network module activity values to distinguish cancer subtypes within the two distinct breast and ovarian cancer patient cohorts in comparison to all genes in the network (network genes) and previously published discriminatory gene sets as described in detail in the main text.

To determine differential module activation between selected sample subclusters we calculated the average of the network module activity for the subcluster of interest and all other samples individually and then computed the differences in average module activity between these sub-groups. To compare the differential activation of network module activity between two data sets we calculated the difference of average network module activity of clusters of interest in each of the data sets individually and then calculated the absolute values of the differences. We then determined the top 15% of differences for each data set individually and selected for further analysis those pathways modules that showed the same trend for higher or lower network module activity within both data sets.

### Sample clustering

We subjected the standardized median network module activity values for complete linkage hierarchical clustering of the patient samples using Pearson correlation as the distance measure. The heatmap-like representation in the hierarchical clustering is achieved through standardization of the data over all samples resulting in a mean gene vector of zero and a standard deviation of one; red and green labels were assigned to values with a higher and lower expression than the mean, respectively. Furthermore, we applied *k*-means clustering as an unsupervised classification method on all patient samples. We tested Spearman and Pearson correlation measures for *k*-means clustering and found that with regard to the ER-status, the Pearson correlation resulted in sample clustering with more significant *p*-values. Therefore, we decided to keep Pearson correlation rather than Spearman correlation for further analysis. In addition to the network module activity value, we used the normalized expression values of all network genes or those genes representing known discriminative gene signatures for hierarchical and *k*-means-based clustering. We determined the optimal number of expected clusters independently of the data labels given by the data sets and found that a cluster number of two is optimal for all four data sets (data not shown). For this cluster stability analysis we used the clValid tool (Brock et al., 2011) and determined the expected number of clusters according to the Silhouette width measure. In order to assess the quality of resulting *k*-means clusterings we assigned each clustering the *p*-value from a chi-square test using the respective known annotations. It is therefore possible to compare the quality of the clustering results.

## Conflict of Interest Statement

The authors declare that the research was conducted in the absence of any commercial or financial relationships that could be construed as a potential conflict of interest.

## Supplementary Material

The Supplementary Material for this article can be found online at http://www.frontiersin.org/Systems_Biology/10.3389/fphys.2013.00124/abstract

## Research Topic:

From structural to molecular systems biology: experimental and computational approaches to unravel mechanisms of kinase activity regulation in cancer and neurodegeneration

## Topic Editor:

Matteo Barberis, Humboldt University Berlin, Germany; Max Planck Institute for Molecular Genetics, Berlin, Germany
